# Polypyrimidine Tract Binding Protein (hnRNP I) Is Possibly a Conserved Modulator of miRNA-Mediated Gene Regulation

**DOI:** 10.1371/journal.pone.0033144

**Published:** 2012-03-09

**Authors:** Bart Engels, Guillaume Jannot, Judit Remenyi, Martin J. Simard, György Hutvagner

**Affiliations:** 1 Wellcome Trust Centre for Gene Regulation and Expression, College of Life Sciences, University of Dundee, Dundee, United Kingdom; 2 Laval University Cancer Research Centre, Hôtel-Dieu de Québec (CHUQ), Québec City, Québec, Canada; 3 Centre for Health Technologies, University of Technology, Sydney, Sydney, Australia; Beckman Research Institute of the City of Hope, United States of America

## Abstract

MiRNAs can regulate gene expression through versatile mechanisms that result in increased or decreased expression of the targeted mRNA and it could effect the expression of thousands of protein in a particular cell. An increasing body of evidence suggest that miRNAs action can be modulated by proteins that bind to the same 3′UTRs that are targeted by miRNAs, suggesting that other factors apart from miRNAs and their target sites determine miRNA-modulation of gene expression. We applied an affinity purification protocol using biotinylated *let-7* miRNA inhibitor to isolate proteins that are involved in *let-7* mediated gene regulation that resulted in an affinity purification of Polypyrimidine Tract Binding protein (PTB). Here we show that PTB interacts with miRNAs and human Argonaute 2 (hAgo2) through RNA as well as identified potential mammalian cellular targets that are co-regulated by PTB and hAgo2. In addition, using genetic approach, we have demonstrated that PTB genetically interacts with *Caenorhabditis elegans let-7* indicating a conserved role for PTB in miRNA-mediated gene regulation.

## Introduction

MicroRNAs (miRNAs) are conserved key regulators of gene expression. They mainly repress protein translation via seemingly distinct mechanisms (reviewed in: [1]) however; recently they were also shown to be involved in enhancing translation at specific cellular environment [2]. miRNAs are essential for proper development in diverse organisms, they are involved in many disease including cancer. Furthermore, in mammals miRNAs alter the expression of thousands of proteins suggesting that they are also responsible for regulating the protein homeostasis in cells by fine-tuning the proteome [3], [4]. miRNAs are incorporated into the RNA induced silencing complex (RISC), in which the core protein an Argonaute family member (reviewed in: [5]). These complexes pair with their targets through the seed sequences that span from 2^nd^ to the 8^th^ nucleotide of the 5′ end of a miRNA. There are increasing amount of evidence that other RNA binding proteins are also involved in modulating miRNA-mediated gene expression at the effector step. HuR, an AU-rich element (ARE) binding protein, was demonstrated to relieve the miR-122 mediated CAT-1 repression in human hepatocarcinoma cells upon amino acid starvation [6]. Another RNA binding protein Dnd1 was shown to protect miR-430 targeted mRNAs in zebrafish primordial cells and miR-372 targeted mRNAs in human cells derived from germ line through binding to U-rich regions (URR) located in the miRNA targeted mRNA regions [7]. CRD-BP (IMP-1) attenuates miR-183-mediated gene silencing by preventing the association of Ago2 complexes with the regulated 3′ UTR [8]. Furthermore, the affinity purification with tagged human Ago2 resulted in the co-purification of a range of RNA binding proteins that have functions in diverse step of RNA biogenesis, transport and RNA translation. Indeed, UPF1 and RBM4 (both associated with hAgo2 and hAgo1) have already been demonstrated to be required for miRNA-mediated gene silencing [9], [10]. Some of these co-factors identified by proteomics could also modulate miRNA-mediated gene expression in a target or miRNA specific manners since RNA was shown to mediate many of these interactions [10].

Polypyrimidine Track Binding protein (PTB), or hnRNP I, is a shuttling RNA binding protein that recognizes short pyrimidine rich sequences and it is involved in the regulation of a wide variety of RNA-dependent biological processes (reviewed in [11]). PTB is a negative and positive regulator of alternative splicing and it regulates its own splicing [12], [13], [14], [15], [16], [17]. PTB could also bind to the 3′UTR of mRNAs and this interaction was shown to be important to regulate mRNA transport and the stability of certain mRNAs [18], [19], [20], [21], [22]. PTB is a key factor in Internal Ribosomal Entry Site (IRES) mediated translation initiation of viral (reviewed in [23]) and cellular mRNAs via its association with the 5′UTRs of these mRNAs [24], [25], [26]. PTB has four RNA recognition motif (RRM) domains and all are capable of binding RNAs [27]. An important structural feature of its interaction with RNA is that RRMs 3 and 4 form a stably packed “back-to-back” didomain, necessitating looping of a stretch of at least 12 nt of RNA between the two pyrimidine motifs recognized by RRMs 3 and 4 [28] [29]. PTB could execute some of its diverse functions by acting as a RNA chaperone and restructuring the RNA so as to either mask, or promote the accessibility of, binding sites for other effector proteins or miRNAs [30]. Interestingly, expression of both PTB and its paralogue nPTB are regulated by miRNAs during neuronal and muscle differentiation, and PTB also regulates expression of its paralogues via splicing [31], [32], [33]. Moreover, PTB can be affinity purified with the conserved loop sequence of the hsa-miRNA-101-1, suggesting a potential role in the regulation of the processing of this miRNA family [34].

Here we have shown that PTB is in complex with human Ago2 and miRNAs. We have also identified potential mRNAs that are co-regulated by PTB and Ago2 post-transcriptionally in human cells. Furthermore, a genetic interaction observed between *C. elegans* PTB and let-7 miRNA supports a conserved function of PTB in modulating miRNA-mediated gene regulation.

## Results

### Affinity purification of PTB using biotinylated 2′-O-Methyl let-7 inhibitor

**Figure 1:**
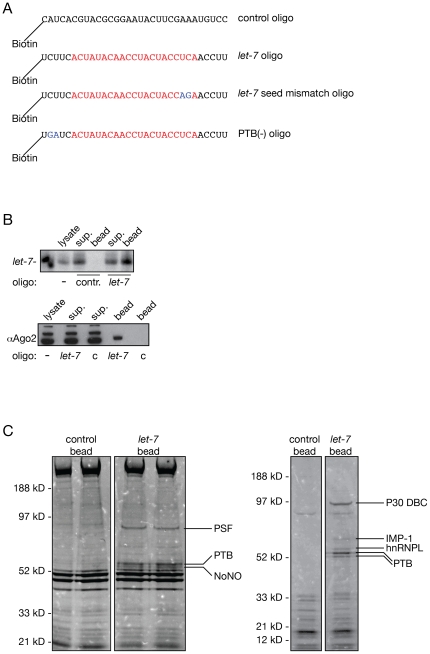
Affinity purification of *let-7* associated complexes. (A) Biotinylated 2-*O*-methylated oligos used in this study. Sequences highlighted with red are complementary to *let-7a*. Blue nucleotides indicate changes generated from the original *let-7* oligo. (B) Northern hybridization (top panel) and Western blot (bottom panel) show that *let-7* oligo specifically purifies *let-7* miRNA and hAgo2 protein. sup.: supernatant; c and cont.: control oligo. (C) Proteins co-purify with *let-7* oligo. Right and left panels show the results of the independent affinity purifications. Proteins that are specifically pulled down with the *let-7* oligo are labeled next to the stained gels.

Inhibitors of miRNAs are widely used *in vitro* and *in vivo* in diverse cells and organisms for investigating miRNA functions and characterizing miRNA-target interactions [35], [36], [37]. These efficient and specific inhibitors are usually modified RNase resistant oligonucleotides with a perfect complementary to their target miRNAs. A 2′-*O*-Methyl containing oligonucleotide inhibitor that interferes with *let-7* function in human cells and *C. elegans* has already been reported (Fig. 1A) [36]. In addition, a biotin tagged version of this oligo pulls down constituents of miRNA complexes [36], [38]. We asked if we could use this approach to purify additional proteins associated with the *let-7* programmed miRNA induced silencing complex (miRISC) in human cells. First, we tested if we could detect *let-7* and human Ago2, the components of the *let-7* programmed minimal RISC, in the bound fraction purified with the biotinylated *let-7* complementary oligo from HeLa cell lysates. The affinity purification showed that both Ago2 and *let-7* were bound to the *let-7* specific oligo but they were not detectable in the bound fraction of the affinity purification carried out with a non-specific 2′-*O*-Methyl oligo (Fig. 1B). Next, we carried out scaled-up affinity purifications to identify proteins that bound specifically along with *let-7* and the *let-7* associated RNPs. We found several proteins that co-purified with the *let-7* complementary oligo, but the only protein that we identified in at least two independent affinity purifications was PTB (Fig. 1C, the two panels show the result of the two independent affinity purifications).

### PTB binding to the let-7 complementary oligo is sensitive to mutations in the let-7 seed complementary sequence

**Figure 2:**
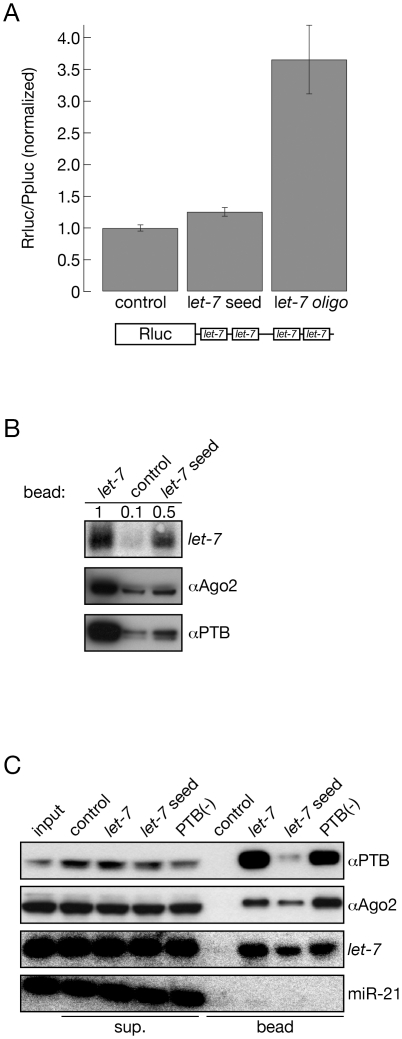
PTB association with the *let-7* bead depends of the *let-7* seed complementary sequences. (A) *let-7* seed mutant oligo could not inhibit *let-7* mediated gene repression. Renilla luciferase expressing plasmid containing a part of the 3′ UTR of human HMGA2 that carries four *let-7* target sites were transfected into HeLa cells together with Firefly expressing plasmid, as internal control, and the indicated 2′-*O*-methyl oligos. The graph shows the result of the dual-luciferase assay normalized to the control oligo. The error bars represent the standard error of three experiments. (B) *let-7*, hAgo2 and PTB are sensitive to the presence of the seed sequence of the *let-7* oligo. The quantity of *let-7* miRNA associated with the indicated oligos was quantified using Northern hybridization and normalized to the amount of miRNA pulled down with the wild-type *let-7* oligo. The presences of hAgo2 and PTB on the indicated beads were monitored by Western hybridization. (C) PTB association with the *let-7* column does not depend on the presence of the canonical PTB site in the oligo. Affinity purifications were carried out with the indicated oligos and the association of miRNAs, hAgo2 and PTB with these oligos was monitored by Northern hybridization and Western blotting. sup.: supernatant.

Since PTB is an abundant RNA binding protein, we asked whether binding of the protein to the column depends on characteristics of the miRNA-target interactions or if it is a non-specific interaction with the 2′-*O*-methyl RNA column. MiRNAs bind their target through the seed sequence; we therefore mutated two nucleotides in the *let-7* oligo that pair with the seed sequence of members of the *let-7* miRNA family (Fig. 1A). We first tested this oligo in miRNA inhibitory study in human cells to see if we were able to abrogate its influence of miRNA-mediated gene regulation. We co-transfected the control, the *let-7* complementary, and the *let-7* seed mismatched oligos into HeLa cells together with a luciferase reporter plasmid that carried a portion of the 3′UTR of the human HMGA2, which contains four bona fide *let-7* target sites [39], [40]. As expected, the *let-7* complementary oligo enhanced the expression of the reporter plasmid significantly by inhibiting the miRNA function (Fig. 2A). On the other hand, the *let-7* mismatched oligo did not show any significant effect on the expression of the *let-7* reporter suggesting that the mutated oligo no longer interferes with miRNA action (Fig. 2A). Next we used the seed mismatched oligo in affinity purification experiments to see how its affinity to the component of the *let-7* programmed miRISC and PTB is affected. Quantification of bound *let-7* showed that the seed mismatched oligo bound only half the amount of *let-7* that was affinity purified with the *let-7* complementary oligo (Fig. 2B). In addition, the introduced seed mismatches significantly reduced the oligo affinity to Ago2 and PTB (Fig. 2C and D). We noticed that the oligo we are using for affinity purification contains a canonical PTB binding motif: UCUUC (Fig. 1A). To determine whether the interaction between PTB and the oligo is mediated by this motif, we generated a new oligo with two mutations in the putative PTB binding site (Fig. 1A: PTB(-) oligo). Affinity purification with this oligo showed similar levels of bound Ago2, PTB and *let-7*, indicating that our purification was indeed dependent upon the *let-7* binding and thus specific (Fig. 2C).

### PTB interacts with the miRNA programmed RISCs in an RNA dependent manner

**Figure 3:**
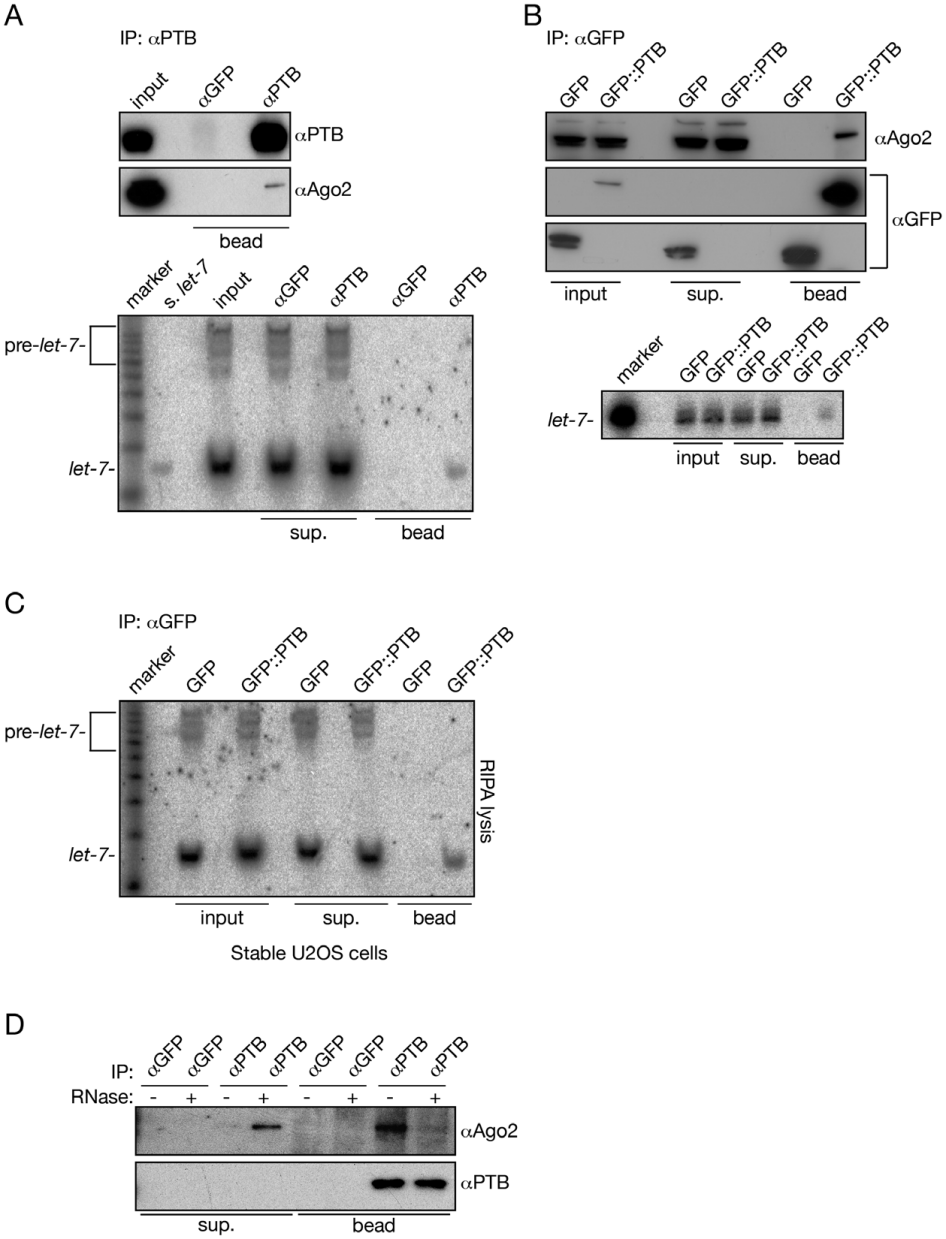
PTB is associated with hAgo2 and *let-7* miRNA. Endogenous PTB in Hela cells (A), PTB fused with GFP in HeLa cells (B) and stably expressed GFP::PTB in U2OS cells (C) co-purify with endogenous hAgo2 and *let-7*. Immunoprecipitations (IP) were carried out with the indicated antibodies. The bound fractions were assayed for hAgo2 and PTB with western blotting (top panels) and for *let-7* with Northern hybridization (bottom panels). (D) PTB association with Ago2 is mediated by RNA. IPs were carried out with antibodies against GFP and PTB. The parts of the bound fraction were subjected to RNase treatment and the supernatants of the RNAse treated beads and the remaining bound fractions were assayed for hAgo2 and PTB by Western blotting.

To confirm the association between PTB and the *let-7* programmed RISC, we first carried out immunoprecipitation experiments with antibodies raised against PTB. We showed that PTB imunoprecipitates with Ago2 (Fig. 3A upper panel) and the mature *let-7* miRNA (Fig. 3A lower panel). In order to test if this interaction is specific, we repeated this experiment using different lysis protocols (Figure S1A and B) and antibodies that recognizes different epitopes of PTB (Figure S1C). In all cases, we could detect Ago2 and *let-7* specifically associated with the PTB bound fractions. Next, we transfected HeLa cells with a GFP::PTB fusion plasmid in parallel with plasmid only expressing GFP and we carried out immunoprecipitation with a GFP specific antibody. We found that Ago2 and *let-7* co-immunoprecipitate with the GFP::PTB but not with GFP alone (Fig. 3B). Finally, we generated U2OS cells constitutively expressing GFP::PTB and repeated the immunoprecipitation . This experiment again showed that *let-7* specifically associated with PTB (Fig. 3C). Finally, we carried out co-fractionation experiment and observed that a substantial fraction of *let-7* co-fractionates with PTB (Figure S2).

Next we asked whether other miRNAs are associated with PTB or its association is specific to *let-7*. When we re-hybridized the RNAs derived form PTB IPs with a probe detecting miR-21, we observed its presence in the PTB containing bound fractions (Figure S3A and B). This data suggest that PTB interacts with diverse miRISCs and it may have a more general role in miRNA-mediated gene regulation.

Next we tested whether PTB binds to RISC solely via protein-protein interaction or if its binding is mediated by RNA. When we RNAse treated the bound fraction of the immunoprecipitated PTB we observed that the majority of Ago2 was released from the bead suggesting that PTB is primarily associated with miRNA complexes via RNAs (Figure 3D).

**Figure 4:**
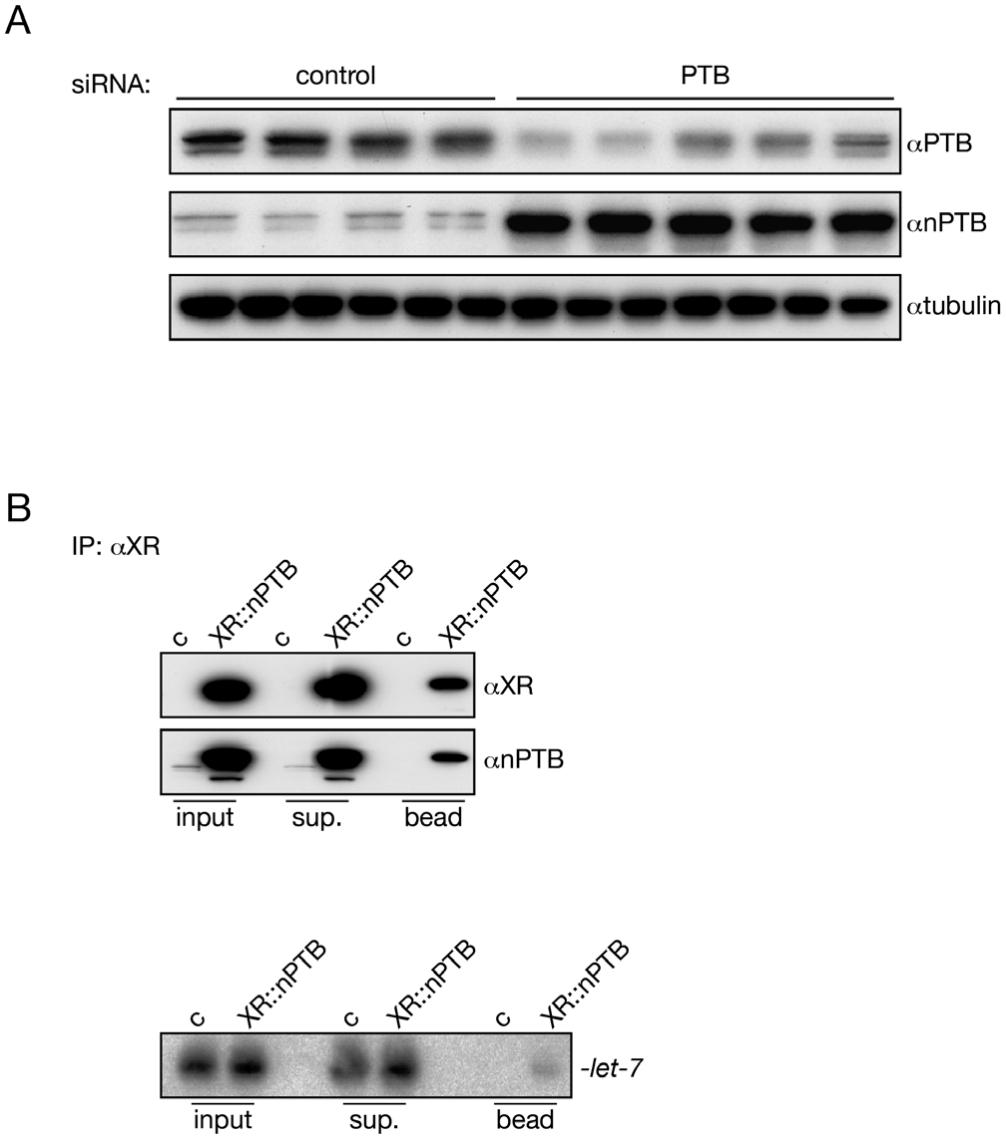
nPTB could also be associated with miRISC. (A) The knock down of PTB results in the increase of nPTB expression in HeLa cells. PTB was knock down with specific siRNA and PTB and nPTB levels were monitored with Western blotting. Tubulin was used as a loading control. *: non-specific hybridization visualized by he nPTB abtibody. (B) nPTB is associated with miRNA. XR tagged nPTB was overexpressed and IP was carried out with antibody recognizing XR. The efficiency of the IP was checked with Western blotting using XR and nPTB antibodies. RNA was purified from the immunoprecipitates and assayed for the presence of *let-7* using Northern blotting. c: empty bead.

Knocking down PTB in HeLa cells results in the upregulation of nPTB, the neuron specific homologue of PTB, that has similar function in the regulation of splicing in HeLa cells [32]. Therefore, we tested if the depletion of PTB could induce nPTB expression and whether nPTB could bind to miRNA complexes. As previously observed, when PTB expression was inhibited with siRNA we also detected a marked increase in nPTB expression [31], [32], [33] (Fig. 4A). We also showed that the XR tagged nPTB co-immunoprecipitates with *let-7* (Fig. 4B). This data suggests that the PTB paralogues might have redundant functions in miRNA-mediated gene regulation.

### PTB alters Argonaute 2 association of human mRNAs

After establishing the connection between PTB and the miRNA loaded RISC, we went to look for mRNAs that are regulated by miRNAs to determine whether PTB could modulate this interaction. We hypothesized that if PTB and miRNAs are involved in the regulation of mRNAs then knock-down of PTB should affect the association of the miRNA-targeted mRNAs with miRISC. Spellman and colleagues reported a 2D DiGE proteomics analysis that identified proteomic changes in response to PTB knock-down in HeLa cells [32]. While many of the observed changes in protein expression were explained by PTB-dependent alternative splicing, some of the up and down-regulated proteins could not be explained by such events. The mRNAs of these proteins are potential candidates for co-regulation by PTB and miRNAs.

**Figure 5:**
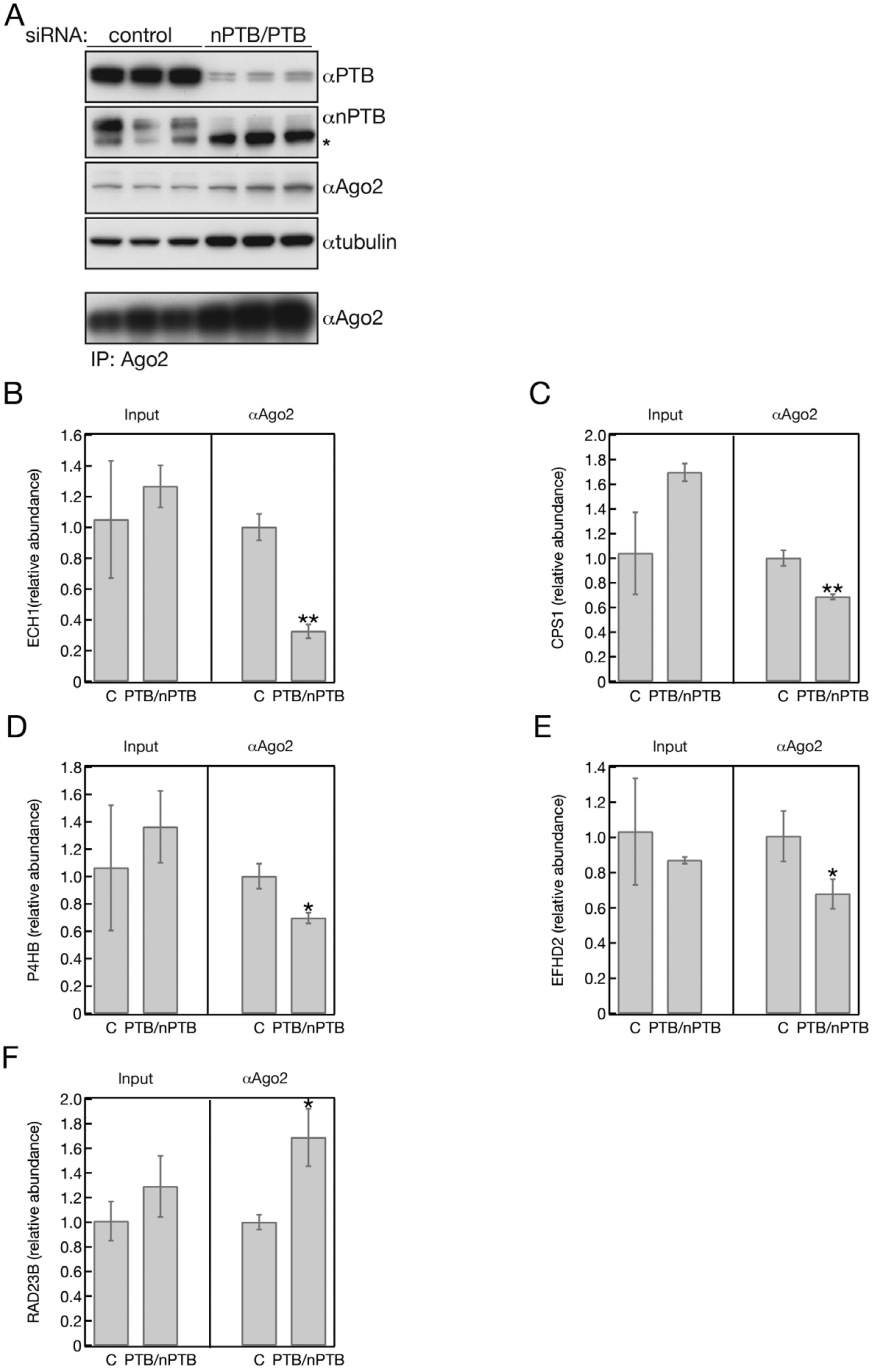
PTB alters Ago2 association of mRNAs in HeLa cells. (A) PTB and nPTB was simultaneously knocked down in triplicates in HeLa cells and Ago2 was immunoprecipitated from control and PTB/nPTB siRNA transfected cells. PTB, nPTB, Ago2 expression was followed by Western hybridization. Tubulin was used as loading control. *: non-specific band detected with the nPTB antibody. (B–F) q-PCR analysis of mRNAs which association with Ago2 is modulated by PTB. RNAs were isolated from control and PTB/nPTB siRNA transfected cells and from Ago2 IPs obtained from the same cells. RNAs were quantified and normalized with GAPDH RNA. The data show the relative abundance of the normalized RNAs compared to the control siRNA transfected cells and the Ago2 IP from the same cells. Error bars represent the standard deviation of three independent experiments (A). *: p<0.05, **: p<0.001.

We therefore knocked down PTB and nPTB expression in Hela cells in triplicates and immunoprecipitated Ago2 from the cells transfected with the control and PTB/nPTB siRNAs (Figure 5A). We next purified RNAs from the Ago2 immunoprecipitates and subjected them to q-PCR using primers that specifically amplify ten candidate mRNAs chosen from the result of the 2D DiGE proteomics. Then, we quantified the changes in the relative abundance (normalized with the level of GAPDH mRNA) of the selected mRNAs in the Ago2 IPs derived from the PTB/nPTB knock down samples by comparing them to the Ago2 IPs were carried out from the control siRNA transfected cells. We also measured the level of the selected mRNAs in total RNAs isolated from the control and PTB/nPTB knock cells. We observed that knocking down PTB/nPTB does not have significant affect on the steady state mRNA levels for most of the selected putative targets (Figure 5 B–F). In the case of five putative targets, we found significant differences in the change of the levels of mRNAs associated with Ago2 after PTB/nPTB knock down suggesting that these mRNAs are co-regulated by PTB and miRNAs post-transcriptionally. In four cases (ECH1, CPS1, P4HB, EFHD2) the relative mRNA levels were decreased in the Ago2 IP after PTB/nPTB knock down, indicating that PTB promotes miRNA binding (Figure 5B–E). These cases are consistent with the preceding observations of an association between PTB and Ago2. In contrast, PTB depletion resulted in an increase of RAD23B mRNA in the Ago2 immunoprecipitates, indicating that in this case PTB antagonizes the miRNA-mediated gene regulation of RAD23B (Figure 5F). The majority of these five mRNAs have conserved miRNA target sites predicted by Targetscan (http://www.targetscan.org) and/or PicTar (http://pictar.mdc-berlin.de) corresponding to miRNAs are expressed in HeLa cells [41]. Also, Rad23B was shown to be targeted by miRNAs in hypoxia [42]. The only exception is ECH1 that only contains non-conserved predicted miRNA target sites. Also, 4 out of 5 potential PTB/miRNA targets (CPS1, EFHD2, P4HB and RAD23) were identified to bind to PTB using PTB iCLIP experiments (J. Ule personal communication) [17].

### PTB modulates let-7 mediated gene silencing in C. elegans

**Figure 6:**
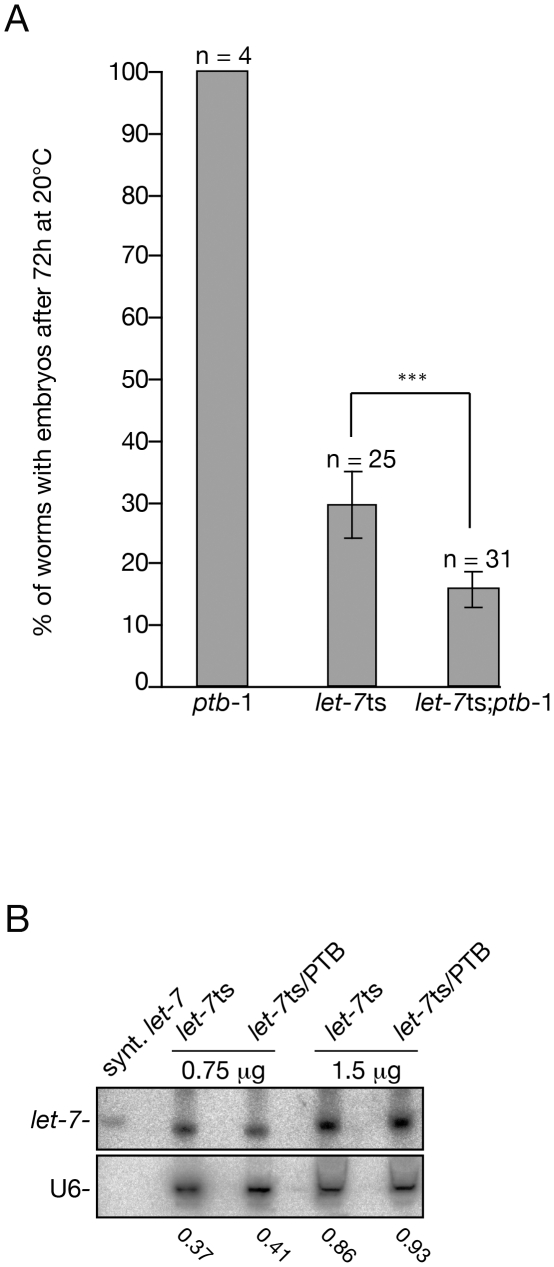
PTB and *let-7* miRNA contribute together to regulate gene expression in *C. elegans*. (A) Synchronized L1 animals were placed at semi-permissive temperature (20°C) and adult animals were scored after seventieth-two hours. The animal sterility observed in the population is caused by either a vulval bursting at the L4-adult transition or by a severe gonadal defect. Error bars represent the 95% confidence interval from independent experiments (n) where between 20 and 40 animals have been scored. ***: p<0.0001 (B) *let-7* level remained unchanged in the *let-7ts*/*ptb-1* animals. RNAs were purified from the indicated genotypes and probed for *let-7* and U6 RNAs. The amount of RNA was used for Northern blotting is indicated on the top of the panel and the U6/*let-7* ratios are presented at the bottom of the panel.

In order to test if the functional interaction between PTB and miRNAs are conserved, we decided to test this interaction in animals using the nematode *Caenorhabditis elegans*. Since *let-7* loss-of-function is lethal [43], we used a *C. elegans* strain that carries a thermosensitive (ts) allele of the *let-7* gene ((*let-7(n2853*)); [43]. We can therefore test if the *C. elegans* ortholog of the human PTB gene called *ptb-1* contributes to *let-7*-mediated gene regulation in animals. While *ptb-1(gk113)* animals has no obvious phenotype, the loss of *ptb-1* in *let-7ts* animals enhanced the observed phenotype (the double mutant population has twice as much sterile animals than the *let-7ts* animals: Figure 6A). Since we did not observe change in the steady state level of *let-7* in the double mutant (Figure 6B), we concluded that PTB is likely required for miRNA-mediated gene silencing at the effector step.

Taken together, our data suggest that like observed in humans, *C. elegans* PTB is working in collaboration with *let-7* miRNA to regulate *let-7*-specific gene.

## Discussion

We have affinity purified PTB with biotinylated miRNA complementary oligonucleotides and showed that PTB/nPTB associate with the miRISC in an RNA dependent manner. This observation suggests that the interruption of the miRNA and the target mRNA with complementary miRNA oligonucleotides does not necessarily results in the full dissociation of the miRISC and the targeted RNA.. It is plausible that the dissociated miRISC is still associated with the targeted mRNA via its binding to the rest of the regulatory complex and/or nearby co-regulatory miRISC and PTB/nPTB also bind to these RNAs.

There is increasing amount of evidence that RNA binding proteins that bind to the 3′UTRs of mRNAs have important roles in regulating miRNA-mediated gene expression. HuR has been shown to relieve the attenuation of gene expression mediated by a specific miRNA in specific cells as a consequence of amino acid starvation [6]. Dnd1, a developmentally regulated RNA binding protein modulates functions of several miRNAs in vertebrates by binding to U-rich sequences of the 3′UTRs and inhibiting the accessibility of miRNA target sites of a subset of mRNAs [7]. It was also demonstrated that IMP-1 binds to the 3′UTR of the βTrCP1 mRNA and it prevents miR-183-medited repression [8]. The affinity purification with human recombinant Argonaute proteins revealed that many RNA binding proteins with functions in diverse steps of mRNA maturation, transport, mRNA stabilization and translation co-purify with hAgo1 and hAgo2. Among them, RBM4 and UPF1 were shown to be required for miRNA-mediated gene regulation [9] [10] The depletion of these two proteins resulted in similar effect that we observed in PTB/nPTB knock down in Hela cells in the majority of the cases. What could be the mechanism by which PTB is involved in miRNA-mediated gene regulation?

Apart from its function as a regulator in splicing PTB has been demonstrated to participate in a wide range of cytoplasmic event by binding to mRNAs (recently reviewed in [11]). PTB is a well-characterized IRES-*trans*-acting-factor (ITAF) that involved in regulating the translation of viral and cellular IRES containing RNAs by binding to the IRES elements in their 5′UTR [24], [25], [26]. PTB also can regulate gene expression by binding to elements in the 3′UTR. For instance, PTB has been demonstrated to stabilize certain mRNAs such as insulin, VEGF, iNOS, CD154, PGK2, also via binding to the 3′UTR of these messages [18], [19], [20], [21], [22]. However, it is very likely that PTB has additional yet uncharacterized functions in the cytoplasm because immunoprecipitation of the cytoplasmic PTB fraction followed by microarray profiling in HeLa cells showed that more then 1600 mRNAs are enriched in the PTB purified complex suggesting a more general role for PTB in the cytoplasm [44]. Interestingly, this study also revealed that the PTB associated transcriptome consist of mRNAs that have significantly longer 3′UTRs which is also a characteristic of miRNA regulated transcripts [45]. In addition, PTB sites have been shown to be enriched in the 3′ UTRs and the coding regions that are also frequently targeted by miRNAs [44], [45]. Moreover, many of the changes in protein expression that occur in HeLa cells upon knock-down of PTB and nPTB cannot be explained by changes in alternative splicing of their mRNAs. [32].

PTB binds to short stretches of pyrimidine-rich sequences with its four RRM domains, and this binding could either directly obstruct binding of other proteins or indirectly facilitate or prevent the binding of other proteins to the RNA by modulating the RNA structure. This restructuring capability of PTB was suggested to be crucial in IRES initiated translation in which PTB creates loops that is necessary to the association of the 40S ribosome. Similar conformational changes, mediated by PTB binding, were implicated in preventing the binding of splicing factors or proteins that destabilize mRNAs. We hypothesize that PTB act as an auxiliary factor that facilitates miRNA-mediated gene regulation by restructuring mRNAs to provide optimal target accessibility for miRNAs. PTB co-immunoprecipitation with miRISCs is mediated by RNA (Fig. 3D) suggesting that PTB could bind first to the miRNA targeted mRNA, thereby promoting an accessible target site conformation for miRISC binding. A similar mechanism has been proposed in the case of hUPF1 since its helicase domain was shown to be required for miRNA-mediated gene regulation [9].

The existence of PTB-like factors has already been envisaged since in some cases the target recognition of miRNAs do not fully agree with the established principles that are the basis of computational approaches that predict miRNA target sites [46], [47], [48]. Furthermore, detailed analysis of the interaction between *C. elegans* miRNA *lys-6* and its mRNA target *cog-1* revealed the importance of the general context of the 3′UTR in miRNA-mediated gene regulation and suggested that miRNA target interactions should be investigated case by case [47]. In vitro studies however: concluded that siRNA loaded RISCs do not need auxiliary factors for target scanning and cleavage if the thermodynamic properties of the siRNA target sites are favorable [49]. Using short target sequences the authors showed that siRNA loaded RISCs could efficiently cleave the target RNA if the target site is less structured. However, miRNA targeted mRNAs characteristically have long 3′UTRs and in this context it is very likely that RNA binding proteins, like PTB, are required to provide such an open structure for efficient targeting [45]. Our genetic experiments in *C. elegans* suggest that at normal miRNA level PTB may be dispensable but when the miRNA level is limited, PTB is required for efficient miRNA-mediated gene regulation probably by providing a structure that is more accessible for targeting.

## Materials and Methods

### Oligonucleotides, siRNAs

Biotinylated 2′-*O*-Methyl oligos were purchased from Dharmacon.

*Let-7* complementary oligo:

5′-biotin-UCUUCACUAUACAACCUACUACCUCAACCUU-3′,

*let-7* seed mismatched oligo:

5′-biotin-UCUUCACUAUACAACCUACUACGAGAACCUU-3′

PTB(-) oligo:

5′-biotin-UGAUCACUAUACAACCUACUACCUCAACCUU-3′

control oligo:

5′-biotin–CAUCACGUACGCGGAAUACUUCGAAAUGUCC-3′

siRNAs to knock down PTB and nPTB expression were purchased from Dharmacon (On target plus, catalog numbers J-003528-06, 07, 08 and 09 and J-021323-09, 10, 11, and 12 respectively) and were used as an equimolar mixture. For simultaneous PTB and nPTB knock down the equimolar mixtures of PTB and nPTB siRNAs were used in a 2∶1 ratio. As a negative control unrelated siRNA was used: 5′-AGGUAGUGUAAUCGCCUUGTT-3′, 5′-CAAGGCGAUUACACUACCUTT-3′.

DNA oligo (Sigma) to detect *C. elegans* U6 RNA: 5′-AATTTGCGTGTCATCCTTGCGCA-3′.

RNA oligonucleotides to detect human and *C. elegans let-7* in Nortern hybridization: 5′-UAUACAACCUACUACCUCAUU-3′, to detect human miR-21: 5′-UCAACAUCAGUCUGAUAAGCUA-3′ synthetic *let-7a*: 5′-UGAGGUAGUAGGUUGUAUAGU-3′ and RNA to detect tRNA-Ile: 5′-UGGUGGCCCGUACGGGGAUCGA-3′ were purchased from Dharmacon and MWG. Decade 10 bp RNA marker (Ambion) was used as additional size marker.

### Antibodies and Western blotting

Primary antibodies were used in this study to detect human PTB: mouse monoclonal Ab (SH54) NA63 (Calbiochem), goat polyclonal Ab sc-16547 (SantaCruz), mouse mAb BB7 (gift from Chris W. Smith, Cambridge, [50]). For detecting human Ago2: rat monoclonal 11A9 (gift from Gunter Meister, [51]), mouse monoclonal Ab 4F9, sc-53521 (SantaCruz). To detect GFP: mouse monoclonal Ab (Roche). To detect human tubulin: mouse monoclonal Ab DM1A (Sigma). Secondary antibodies were peroxidase conjugated (Jackson Immuno Research). Western blotting was performed by following standard protocols, using gel cassettes (Invitrogen) or the NuPage system (Invitrogen). Proteins were electroblotted and detected either with Thermo Scientific Supersignal West Chemoluminescent substrate or with Millipore Immobilon Western chemonluminescent HRP substrate.

### Plasmids

The following plasmids were used in this study: PTB::GFP, XR::nPTB (gifts from Chris Smith), pEGFP (Clontech). Firefly luciferase: PGL2. Renilla luciferase reporter constructs: pRL-TK H2-H5: Renilla luciferase that containing four *let-7* target sites in the 3′UTR in the context of the part of HMGA2 3′UTR , pRL-TK ΔH2-ΔH5: same as H2-H5 only the seed sequences complementary sites of the *let-7* were mutagenized at second and third nucleotides.

### Cell culture, transfection and cell lysis

Stable GFP and GFP::PTB expressing cell lines were generated by transfection of U2OS (gift from S. Rocha WT centre for GRE, Dundee University) cells with Effectene (Qiagen) and selection using 400 µg/ml of G418. Single colonies were picked after limited trypsinisation and checked for expression by immunofluorescence and Western blotting.

Transfections were performed with Effectene (Qiagen), Lipofectamine 2000 (Invitrogen) and oligofectamine (Invitrogen) and Lipofectamine RNAiMAx (Invitrogen) according to the manufacturers instructions.

The following cell lysis protocols were used in this study: NP40: 50 mM Tris pH 7.5, 150 mM NaCl, 1% NP40, RIPA: 50 mM Tris pH 8.0, 150 mM NaCl, 1% NP40, 0.5% sodium deoxycholate, 0.1% SDS, Polysome buffer: 0.5% NP40 alternative, 130 mM KCl, 10 mM MgCl_2_, 2.5 mM DTT. Mammalian Cell lysis buffer (MCLB): 50 mM Tris pH 7.4, 150 mM NaCl, 1% Triton X100, 1.25 µl/ml beta mercaptoethanol and 0.9 g/ml sucrose All buffers were supplemented with Complete protease inhibitor (Roche) and RNasin (Promega).

### Affinity purifications with biotinylated 2′-*O*-Methyl oligonucleotides

Ten to fifteen 10 cm dishes of HeLa cells (gift from A. I. Lamond laboratory, WT Centre for GRE, Dundee University), (∼80–90% confluent) were lysed in NP40 lysis buffer. 25 µl streptavidin magnetic beads (Dynal, Invitrogen) were equilibrated with 2× Binding buffer (10 mM Tris, pH 7.5, 1 mM EDTA, 2.0 M NaCl) by washing the beads with three times. Beads were resuspended in 100 µl 1× Binding buffer and 100 pMol biotinylated 2′-*O*-Methyl oligonucleotides were added and incubated for two hours in the cold room with gentle agitation. After incubation the beads were washed three times with 1× Binding buffer and two times with lysis buffer. 500–1000 µl lysate were added and incubated on 37C° for one hour. The beads were washed three times with lysis buffer and resuspended in 50 µl lysis buffer. RNA was isolated with 2× PK buffer (200 mM Tris pH 7.5, 25 mM EDTA, 300 mM NaCl, 2% SDS, 2 µg/µl proteinase K) followed by phenol extraction and ethanol preciptitation and monitored with Northern hybridization. For protein assay the washed beads were boiled with the SDS loading dye and the eluate was subjected to Western blotting.

### Imunoprecipitations

Antibodies were bound to Dynabeads protein G (Dynal, Invitrogen) and washed with lysis buffer. Cells were lysed with RIPA or MCLB buffers, incubated overnight with the bound antibodies and were washed with lysis buffers. RNA was isolated with 2× PK and proteins were eluted by boiling the bound fraction in SDS loading buffer and analyzed by Western blotting.

### Gel filtration

Gel filtration was performed on an Akta Explorer machine using a Sepachryl-S300 column (Amersham) in a 4°C cabinet. Cells were lysed in: 50 mM Tris pH 7.9, 12.5 mM MgCl_2_, 10% glycerol, 0.015% NP40 and 150 mM KCl. Fractions were collected and RNA and protein were isolated with 2× PK buffer and monitored with Northern or Western blotting respectively. Low molecular weight marker (Amersham) was used to calibrate the elution profile. The quantity of *let-7* of the fractions was determined using 1 and 10 femtomole *let-7* standards run alongside the fractions on Northern blots.

### Northern hybridizations

Standard Northern blotting for the detection of small RNAs were performed as described [52]. BasIP-MS-2040 imaging plates (Fujifilm) were scanned with FLA-5100 (Fuji) and the data were quantified using ImageGauge v4.21 software.

### *C. elegans* protocol

The *ptb-1 (gk113)* and *let-7ts (n2853)* strains were obtained from the *C. elegans* Gene Knockout Consortium. *C. elegans* experiments were performed at 20°C during the indicate time. For survival analysis, a population of *let-7ts (n2853)*; *ptb-1 (gk113)*, *let-7ts (n2853)* and wild-type N2 animals were synchronized as L1 larvae, grow on plates and scored for % of survival adult animals with embryos after 72 hours at semi-permissive temperature (20°C).

## Supporting Information

Figure S1 **PTB co-purifies with the** ***let-7*** **loaded RISC.** PTB association with the *let-7* loaded human RISC is maintained by using different lysis protocols (A) and; using different antibodies of hAgo2 and PTB (B). The bound fractions of the PTB immunoprecipitates were assayed for hAgo2 and PTB with Western blotting (top panels) and for *let-7* with Northern hybridization (bottom panels). (C) GFP tagged PTB also immunoprecipitates endogenous hAgo2 (top panel) and *let-7* miRNA (bottom panel).
(TIF)

Figure S2 **PTB and** ***let-7*** **co-fractionate in human cells.** Total cell lysate was fractionated through Sephacryl S-300 column. Every second fraction was subjected to RNA and protein isolation. *Let-7* and PTB was monitored with Northern hybridization and Western blotting respectively.
(TIF)

Figure S3 **miR-21 is associated with PTB.** Endogenous PTB in Hela cells (A), and stably expressed GFP::PTB in U2OS cells (B) co-purify with miR-21. Experiments were carried out as it was described at Figure 3. and the Northern hybridizations were repeated with radioactively labeled probe recognizing miR-21.
(TIF)
