# Supplementary figures and images for: Polypyrimidine Tract Binding Protein (hnRNP I) Is Possibly a Conserved Modulator of miRNA-Mediated Gene Regulation
Source: PLoS One. 2012 Mar 9;7(3):e33144. doi: 10.1371/journal.pone.0033144 (PMC3302860; doi:10.1371/journal.pone.0033144)

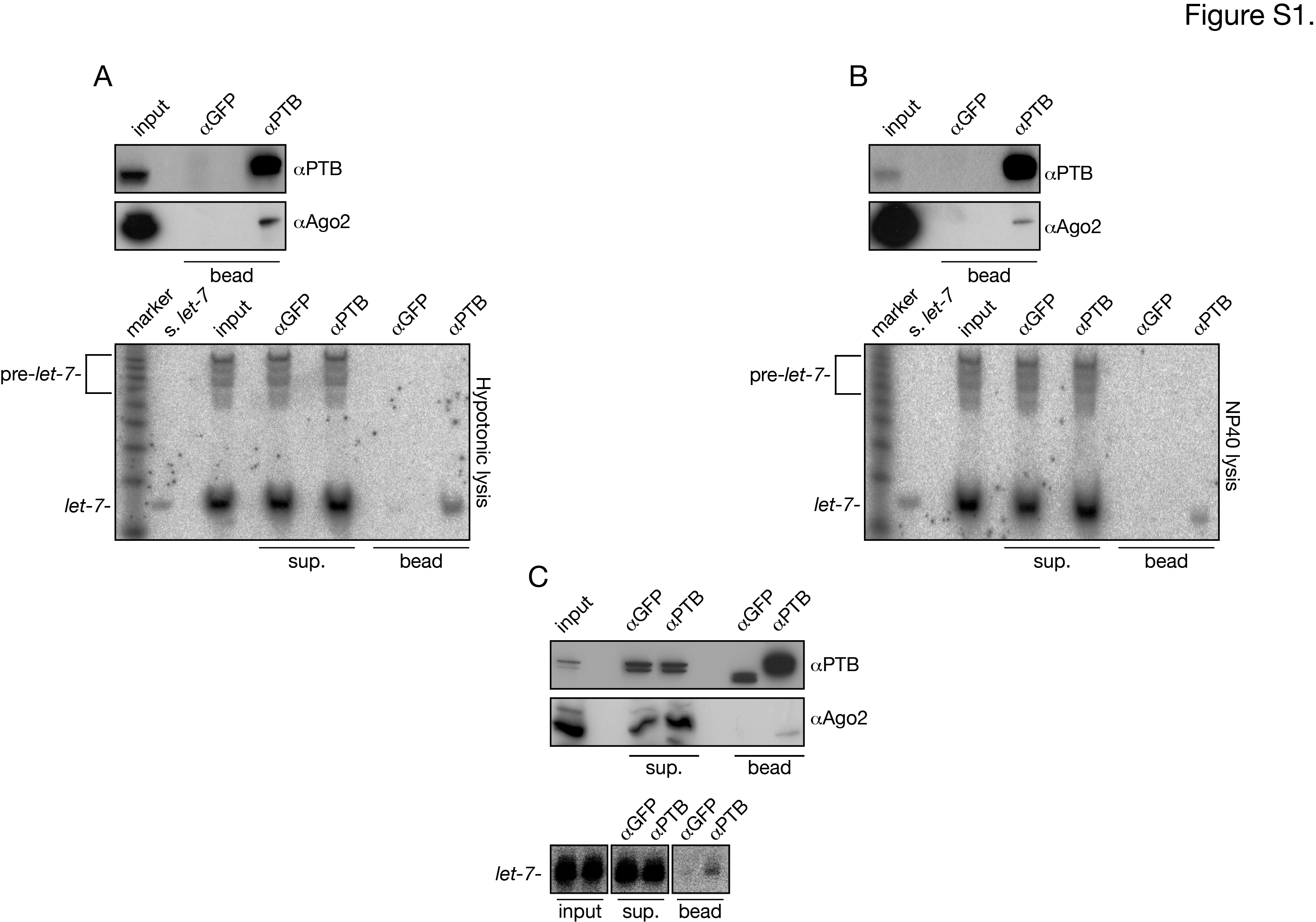

Supplement: Figure S1 — PTB co-purifies with the let-7 loaded RISC. PTB association with the let-7 loaded human RISC is maintained by using different lysis protocols (A) and; using different antibodies of hAgo2 and PTB (B). The bound fractions of the PTB immunoprecipitates were assayed for hAgo2 and PTB with Western blotting (top panels) and for let-7 with Northern hybridization (bottom panels). (C) GFP tagged PTB also immunoprecipitates endogenous hAgo2 (top panel) and let-7 miRNA (bottom panel). (TIF) [file pone.0033144.s001.tif]

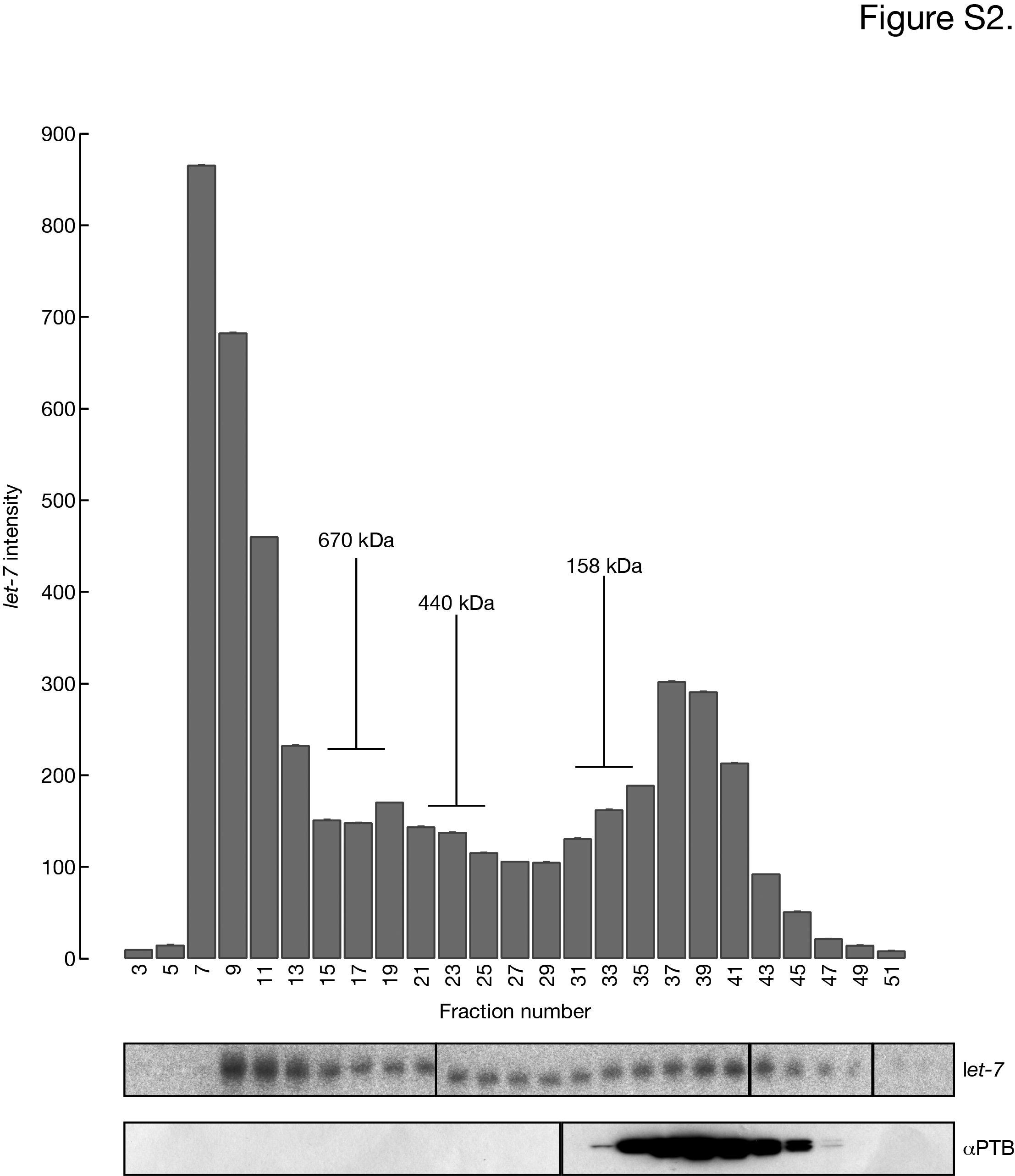

Supplement: Figure S2 — PTB and let-7 co-fractionate in human cells. Total cell lysate was fractionated through Sephacryl S-300 column. Every second fraction was subjected to RNA and protein isolation. Let-7 and PTB was monitored with Northern hybridization and Western blotting respectively. (TIF) [file pone.0033144.s002.tif]

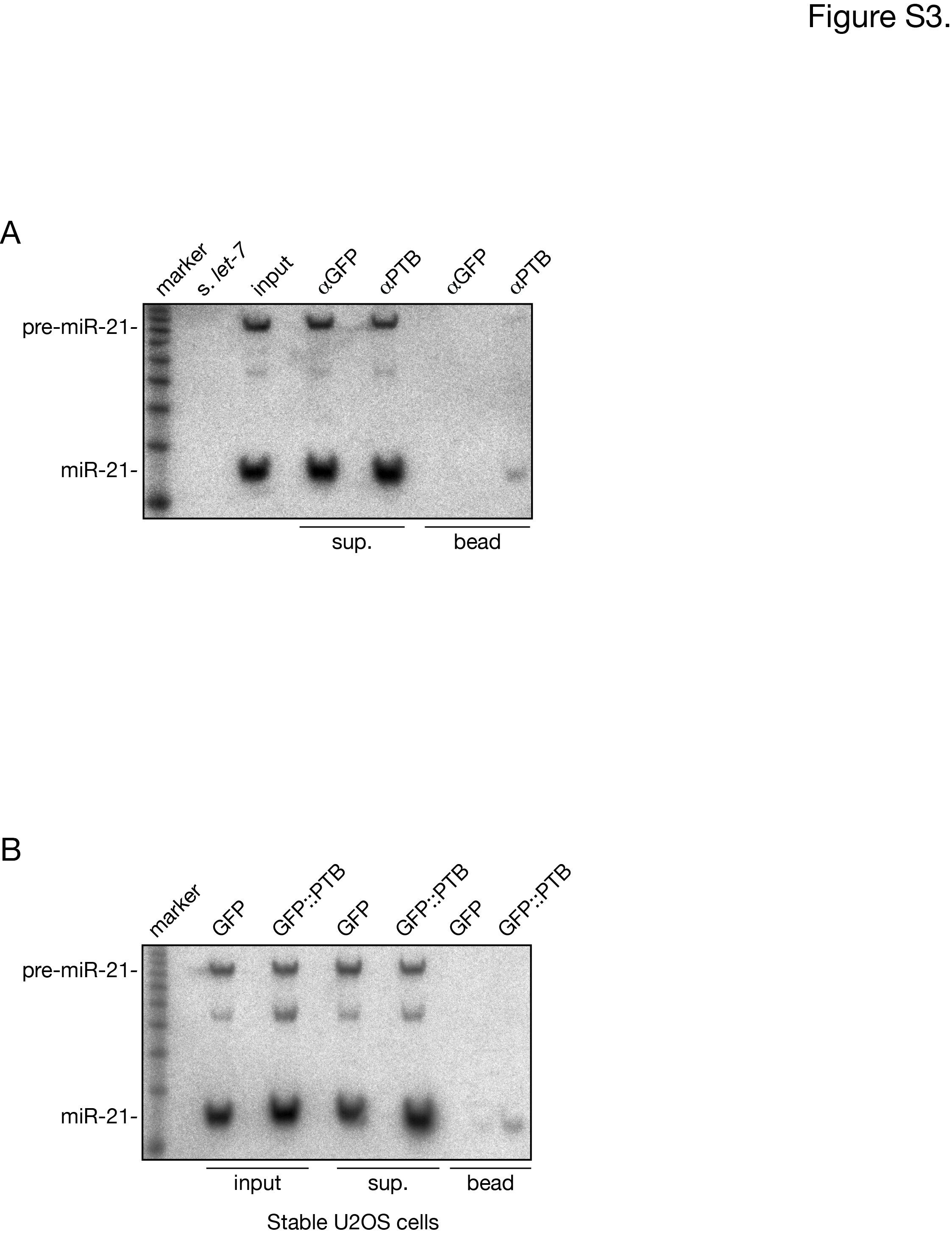

Supplement: Figure S3 — miR-21 is associated with PTB. Endogenous PTB in Hela cells (A), and stably expressed GFP::PTB in U2OS cells (B) co-purify with miR-21. Experiments were carried out as it was described at Figure 3. and the Northern hybridizations were repeated with radioactively labeled probe recognizing miR-21. (TIF) [file pone.0033144.s003.tif]
